# Nuclear receptor binding SET domain protein 1 promotes epithelial-mesenchymal transition in paclitaxel-resistant breast cancer cells via regulating nuclear factor kappa B and F-box and leucine-rich repeat protein 11

**DOI:** 10.1080/21655979.2021.2009963

**Published:** 2021-12-14

**Authors:** Yi Chen, Weiwei Tang, Xuedan Zhu, Lele Zhang, Yinxing Zhu, Hua Xiao, Jin Xu, Yueyu Fang, Xiao Li, Cuiju Tang, Junfeng Shi

**Affiliations:** a Department of Oncology, Nanjing Pukou Central Hospital, the First Affiliated Hospital of Nanjing Medical University, Nanjing, Jiangsu, China; b Hepatobiliary/Liver Transplantation Center, The First Affiliated Hospital of Nanjing Medical University, Key Laboratory of Living Donor Transplantation, Chinese Academy of Medical Sciences, Nanjing, Jiangsu, China; c Department of Oncology, Nanjing First Hospital, Nanjing Medical University, Nanjing, Jiangsu, China; d Department of Nuclear Medicine, Nanjing First Hospital, Nanjing Medical University, Nanjing, Jiangsu, China; e Department of General Surgery, Nanjing First Hospital, Nanjing Medical University, Nanjing, Jiangsu, China; f Department of Thyroid and Mammary Gland Surgery, Nanjing First Hospital, Nanjing Medical University, Nanjing, Jiangsu, China

**Keywords:** Breast cancer, paclitaxel, NSD1, FBXL11, NF-KB

## Abstract

Breast cancer (BC) is regarded as the major cause of cancer-associated deaths in women. Paclitaxel exerts a critical impact on the chemotherapy of BC, but the resistance to paclitaxel becomes a great obstacle in treating the disease. It is reported that noncoding RNA nuclear receptor binding SET domain protein 1 (NSD1) plays a significant role in drug resistance; however, the special role of NSD1 in paclitaxel-resistant BC is unclear. Human BC cell line MCF-7 was used to establish paclitaxel-resistant BC cells (MCF-7/PR). Reverse transcription quantitative polymerase chain reaction (RT-qPCR) displayed that NSD1 and F-box and leucine-rich repeat protein 11 (FBXL11) were highly expressed in BC tissues. Western blotting was utilized for protein level assessment. Cell counting kit-8 (CCK-8), Transwell, wound healing assays, and animal experiments were conducted to examine the influence of NSD1 or FBXL11 on the malignant behaviors of BC *in vitro* and *in vivo*, respectively. Transfected MCF-7/PR cells were injected subcutaneously into BALB/c nude mice with or without treatment of paclitaxel. The nuclear factor kappa B (NF-kB) activity was evaluated by the luciferase reporter assay. Results showed that NSD1 knockdown inhibited the epithelial-mesenchymal transition (EMT), migration and invasiveness of BC *in vitro*, which was rescued by FBXL11 overexpression. Furthermore, NSD1 silencing promoted paclitaxel sensitivity of paclitaxel-resistant BC cells and suppressed tumor growth and paclitaxel resistance *in vivo*. NSD1 knockdown reduced NF-kB activity, while FBXL11 inhibition markedly increased NF-kB activity. Collectively, NSD1 facilitates the EMT, migration and invasion in paclitaxel-resistant BC cells via regulating NF-kB and FBXL11.

## Introduction

Breast cancer (BC) is the most prevalent malignancy among women, resulting in approximately 25% of the cancer-related deaths worldwide [1,2]. At the advanced stage, cancer cells are prone to distant metastasis and affect multiple organs in the body, seriously threatening the life of patients [3]. Over the years, great achievements have been made in the diagnosis and treatment of BC and paclitaxel chemotherapy is one of the most commonly adopted approaches for patients with BC [4]. Paclitaxel is a member of the taxane family that exerts cytotoxic effects on cancer cell [5]. Owing to this function, paclitaxel is regarded as a widely accepted anticancer agent applied in the treatment of a range of cancers including BC [6]. However, the resistance of BC to paclitaxel treatment becomes the main cause of the failure of clinical applications [7,8]. Hence, it is urgent to identify efficient markers for treating paclitaxel-resistant BC.

The nuclear receptor binding SET domain protein 1 (NSD1) is a key gene of the NSD gene family, and its important functional domain SET can catalyze the methylation of H3K36 [9]. Aberrant expression of NSD1 in diverse cancers has been revealed, which is related to tumorigenesis, survival and chemoresistance. For example, in pancreatic ductal adenocarcinoma, high level of NSD1 is significantly related to high clinical stage [10]. Additionally, NSD1 knockout is reported to inhibit the proliferative, migratory, and invasive abilities of hepatocellular carcinoma cells [11]. It was suggested that the head and neck cancer patients with mutations in NSD1 have an improved prognosis and high level of NSD1 renders the resistance of head and neck cancer cells to chemotherapy [12]. This suggests that NSD1 might be a potential biomarker for drug resistance in tumors. However, the function and mechanism of NSD1 in paclitaxel-resistant breast cancer remain obscure.

F-box and leucine-rich repeat protein 11 (FBXL11), also known as KDM2A, is a lysine demethylase [13]. Dysregulation of FBXL11 has been reported to exert important effects on the biological processes of various cancers. For example, FBXL11 suppresses proliferation and angiogenesis in multiple myeloma cells through ubiquitination by targeting 6-phosphofructo-2-kinase/fructose-2,6-biphosphatase 3 [14]. Additionally, FBXL11 regulates the phosphatidylinositol 3-kinase pathway and reverses epithelial-mesenchymal transition to facilitate ovarian cancer progression [15]. Furthermore, it has been validated that there exists a negative feedback loop between FBXL11 and nuclear factor kappa B (NF-kB), whose abnormal regulation contributes to the growth, metastasis and invasiveness in a variety of cancers [16]. Importantly, it was reported that overexpression of FBXL11 decreases the methylation of p65, while overexpression of NSD1 greatly enhances the methylation of p65, suggesting that NSD1/FBXL11 pair of methylase/demethylase enzymes reversibly regulates NF-kB [17]. Nevertheless, the specific relationship among NSD1, FBXL11 and NF-kB in BC needs to be illustrated.

Epithelial-mesenchymal transition (EMT) is a complicated biological procedure where cancer cells are transdifferentiated from an epithelial condition into a motile mesenchymal condition [18]. Importantly, studies have shown that EMT is a major event motivating migration and invasiveness of tumor cells [19–21]. Studies on the EMT procedure in BC have been carried out [22–24]. However, the impact of NSD1 on EMT in BC is still unclear.

This study aimed to reveal the effects of NSD1 on cell migration, invasion, EMT and paclitaxel sensitivity of paclitaxel-resistant BC as well as on tumor growth and EMT *in vivo*. The potential mechanism of NSD1 was also investigated. It was hypothesized that silencing NSD1 may promotes the drug-sensitivity of paclitaxel-resistant BC by interacting with other molecules. Our findings might provide a new perspective for treating patients with BC.

## Materials and Methods

### Tissue specimens

Twenty pairs of BC tissues and matched nontumor tissues were collected from Nanjing First Hospital, Nanjing Medical University (Jiangsu, China). The patients with BC were female who were sensitive to paclitaxel. Age of the patients ranged from 24 to 76 years and the mean age was45.2 ± 9.2 years. The inclusion criteria were as follows: 1) BC diagnosed by histopathological examination; 2) no previous malignancy history; 3) first diagnosis and no radiotherapy or chemotherapy received before admission. The exclusion criteria: 1) age younger than 18 years; 2) patients with multiple clinical disorders besides BC; 3) patients who were transferred from other hospital There were two cases of stage I, four cases of stage II, eight cases of stage III, and six cases of stage IV. Among these patients, distant metastasis occurred in eight cases. All patients were provided with written informed consent. The tissues were rapidly frozen in liquid nitrogen and preserved at −80°C [25]. This study was authorized by the Ethics Committee of Nanjing First Hospital, Nanjing Medical University (Jiangsu, China) and in compliance with the requirements of the Declaration of Helsinki.

## Cell culture

BC cells MCF-7 were obtained from China Cell Culture Center (Shanghai, China). For the establishment of paclitaxel-resistant MCF-7 (MCF-7/PR), MCF-7 cell lines was continually incubated in incrementally increasing concentration of paclitaxel for more than 6 months. MCF-7 and MCF-7/PR cell lines were cultured in Dulbecco’s Modified Eagle’s Medium (DMEM, Gibco, Carlsbad, CA, USA) supplemented with 10% fetal bovine serum (FBS, Gibco), 100 U/mL penicillin (Sigma-Aldrich, USA) and 100 μg/mL streptomycin (Sigma-Aldrich) [26]. All cells were then maintained in a humidified incubator at 37°C with 5% CO_2_.

## Cell transfection

For the downregulation of NSD1 or FBXL11, short hairpin RNA (shRNA) specifically targeting NSD1 (sh-NSD1) or FBXL11 (sh-FBXL11) and the negative control were bought form GenePharma (Shanghai, China). The pcDNA3.1/FBXL11 and control pcDNA3.1 plasmids were obtained from Invitrogen (Carlsbad, CA, USA) to upregulate the expression of FBXL11. All the plasmids were transfected into BC cells with Lipofectamine 2000 (Invitrogen) [27]. RT-qPCR was carried out to analyze the transfection efficiency after 48 h. Sequences of NSD1 and FBXL11 specific-shRNAs in Table 1.

**Table 1.** Sequences of NSD1 and FBXL11 specific-shRNAs

Gene	Sequence (5ʹ→3ʹ)
sh-NC	CGATTGAGTAACTGGAAGATTTTCAAGAGAAATCTTCCAGTTACTCAATCGTTTTTT
sh-NSD1	GCAGATGTAGATTCTGAAATGTTCAAGAGACATTTCAGAATCTACATCTGCTTTTTT
sh-NC	AGACAAACCTACAATCAGCATTTCAAGAGA ATGCTGATTGTAGGTTTGTCTTTTTTT
sh-FBXL11	GCACACCAACAAATATAATGCTTCAAGAGAGCATTATATTTGTTGGTGTGCTTTTTT

## Cell counting kit‐8 (CCK‐8) assay

Cell viability was evaluated by CCK-8 assay. Briefly, MCF-7 or MCF-7/PR cells with or without transfection were seeded into 96-well plates (1 × 10^4^ cells/well) and incubated for 24 h. Paclitaxel was diluted to a certain concentration range (1–1000 nM) in DMEM containing 2% FBS before placed into the plates. After exposure to paclitaxel for 48 h, CCK-8 reagent (5 mg/mL; Solarbio, Beijing, China) was added to the plates and incubated for further 2 h. The relative cell viability was assessed by measuring the optical density at 450 nm with a microplate reader (Bio‐Rad, Hercules, CA, USA) [28].

## Reverse transcription quantitative polymerase chain reaction (RT-qPCR)

Total RNA from BC tissues and BC cells was extracted by TRIzol® reagent (Invitrogen) and subjected to reverse transcription using PrimeScript RT reagent kit (Takara, Japan) to synthesize complementary DNA (cDNA). The RT-qPCR was implemented using SYBR® Premix Ex Taq^TM^ II reagent kit (RR820A, Takara) and an ABI7500 real-time qPCR system (7500, ABI Company, Oyster Bay, NY, USA). Quantification of mRNA levels was performed with the 2^−ΔΔCt^ method, normalized to glyceraldehyde-3-phosphate dehydrogenase (GAPDH) [29].

Primer sequences are as follows:

NSD1:

Forward-5ʹ-AAACTCGGAGGGTGCT-3ʹ

Reverse-5ʹ-CCTGAGGCGTTTCTTCT-3ʹ

FBXL11:

Forward-5ʹ-ACCATCCCCATTACGAAGCC-3ʹ

Reverse-5ʹ-ACACCACACTCTCCTTGCAC-3ʹ

GAPDH:

Forward-5ʹ-TATGATGATATCAAGAGGGTAGT-3ʹ

Reverse-5ʹ-TGTATCCAAACTCATTGTCATAC-3ʹ

## Western blotting

Proteins were collected from BC cells with radio-immunoprecipitation assay lysis butter (Beyotime, Shanghai, China) and measured by the bicinchoninic acid protein assay kit (Beyotime) [30]. Cell proteins were subjected to 10% sodium dodecyl sulfate polyacrylamide gel electrophoresis and transferred to polyvinylidene fluoride (PVDF) membranes (Millipore, Billerica, MA, USA). The membranes were blocked with 5% defatted milk and incubated with following primary antibodies: anti-NSD1 (sc-130,470; 1:500, Santa Cruz Biotechnology, Santa Cruz, CA, USA), anti-β-Actin (ab179467, 1:5000, Abcam Inc., Cambridge, MA, USA), anti-GAPDH (ab8245, 1:6000, Abcam), anti-E-cadherin (ab1416, 1:50, Abcam), anti-N-cadherin (ab76011, 1:5000, Abcam), anti-Vimentin (ab92547, 1:5000, Abcam), anti-FBXL11 (ab191387, 1:1000, Abcam), anti-p65K218me, anti-p65K221me2, and anti-p65 (ab32536, 1:5000, Abcam) overnight at 4°C, and then incubated with the specific secondary antibodies (Abcam Inc., Shanghai, China) at room temperature for 1 h. After several washing, the signal of protein bands was visualized utilizing ECL luminol reagent (Millipore) and quantified by Amersham Imager 600 system (AI600, USA).

## Wound healing assay

BC cells (1 × 10^6^ cells/well) were seeded into 6-well plates and cultured in DMEM containing 10% FBS. Wounds were created on the cell monolayer by making artificial scratches with plastic 10 μL pipette tips. Subsequently, plates were rinsed twice with phosphate buffer saline (PBS). The wound closure was photographed respectively at 0 and 48 h with an inverted microscope at ×40 magnification (Olympus, Tokyo, Japan) [31]. The rate of wound healing was quantified by ImageJ V1.8.0 software (NIH, Bethesda, Maryland) as: (prime wound area-current wound area)/ (prime wound area).

## Transwell assay

Transwell chambers (Corning Inc., Corning, NY, USA) was used for the assessment of the migratory ability of BC cells [31]. After 48 h of incubation, the cells were collected, rinsed with PBS and resuspended in DMEM without FBS. The upper chambers were added with 100 μL of suspension containing 5 × 10^4^ cells. The lower chambers were added with a 24-well plate covered with 500 μL DMEM containing 10% FBS. Twenty-four h later, cells in the upper chambers were removed, and the migratory cells were fixed in 4% paraformaldehyde, stained with 0.5% cystal violet, washed with PBS and photographed with an inverted microscope (Olympus). The invasive ability of BC cells was measured similiar to the above migration assay, apart from that Matrigel (BD Biosciences, San Jose, CA, USA) was precoated for the chambers.

## Luciferase reporter assay

The NF-kB luciferase construct (pNFkB-luc) was purchased from Beyotime. The pNFkB-luc plasmids were co-transfected with sh-NSD1, sh-FBXL11 or sh-NC into BC cells by Lipofectamine 2000 (Invitrogen) [32]. Dual-Luciferase Reporter Assay System (Promega, Madison, Wisconsin, USA) was utilized for measuring the luciferase activity of NF-kB following the instructions of the manufacturer.

## *In vivo* xenograft experiments

Female BALB/c nude mice (4-6-week-old) were obtained from the Shanghai Experimental Animal Center of Chinese Academic of Sciences (Shanghai, China) and divided into four groups (n = 3 per group): sh-NC, sh-NSD1, sh-NC + paclitaxel, sh-NSD1 + paclitaxel. BALB/c nude mice were injected subcutaneously with MCF-7/PR cells (1 × 10^6^ per injection) with transfection of sh-NSD1 or sh-NC. Severn days after cell transplantation, mice were administrated intraperitoneally with paclitaxel (15 mg/kg; Sigma, St Louis, MO, USA) every week. Tumor volumes were quantified every week after being evidently observed and computed by the formula: volume = ½ (length × width^2^) [33]. Four weeks later, the mice were sacrificed under anesthesia. The weight of tumors was measured, and the lungs were collected to evaluate the number of pulmonary metastatic nodules. All procedures were approved by the Institutional Animal Care and Use Committee of Nanjing First Hospital, Nanjing Medical University (Jiangsu, China).

## Statistical analysis

Statistical data were analyzed with SPSS 18.0 statistic software (IBM, NY, USA) [34]. Each experiment was conducted at least three times and data are presented as mean ± standard deviation (SD). Student’s *t*-test was applied to analyze differences between two groups and ANOVA (analysis of variance) followed by Tukey’s *post hoc* analysis was for differences among three or more groups. The criterion of statistical significance was *p* < 0.05.

## Results

This study aimed to investigate the effects of NSD1 on paclitaxel-resistant BC *in vitro* and *in vivo*. We explored the impacts of NSD1 on BC cell migration, invasiveness, EMT, and paclitaxel sensitivity, as well as its potential regulatory mechanism. Additionally, the effect of NSD1 on tumor growth and EMT of BC were also explored. The results indicated that NSD1 exerted its effects on the malignant behaviors of paclitaxel-resistant BC cells by regulating FBXL11 and NF-kB. Moreover, NSD1 also had an impact on tumor growth, EMT, and paclitaxel sensitivity of BC *in vivo*.

## NSD1 is overexpressed in BC and related with a poor prognosis

**Figure 1.**
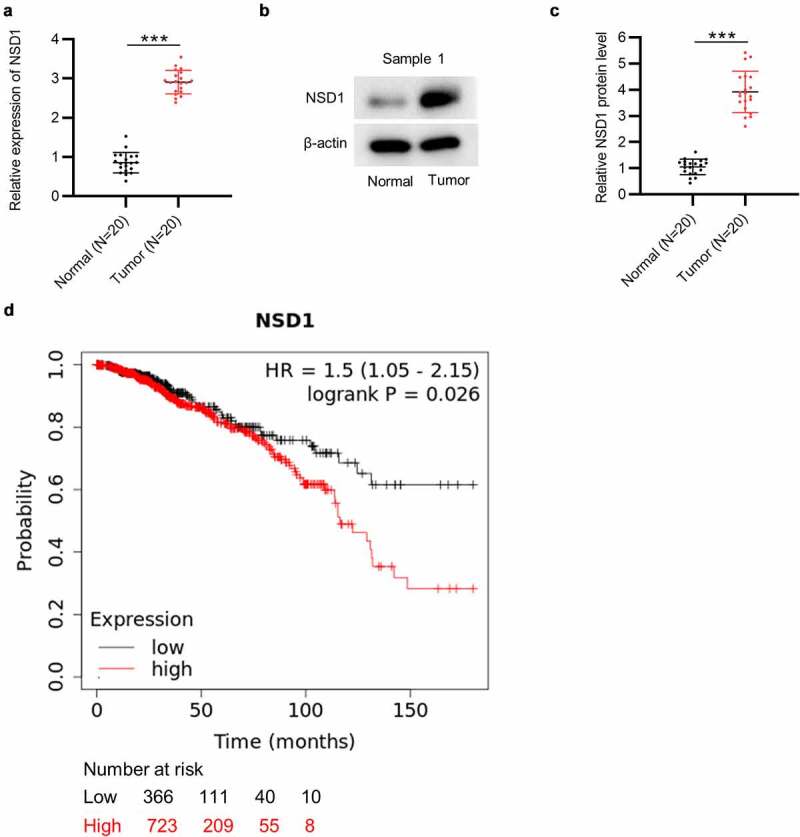
**NSD1 displays high level in BC and is related to poor prognosis**. (a) RT-qPCR of NSD1 expression in 20 pairs of BC tissues and normal tissues. (b-c) Western blotting of the protein expression of NSD1 in 20 pairs of BC and normal tissues. Protein bands of the other 19 samples are provided in the supplementary file named Figure S1. (d) Kaplan-Meier Plotter analysis of the relationship between NSD1 level and BC patient prognosis. ***p < 0.001

The results of RT-qPCR and Western blotting showed that the mRNA level and protein expression of NSD1 were higher in BC tissues than those in matched nontumor tissues (N = 20 pairs) (Figure 1(a-c)). Moreover, the data of Kaplan-Meier Plotter indicate that BC patients with a higher level of NSD1 have a poorer prognosis than those with a lower level of NSD1 (Figure 1(d)). These results suggested that high level of NSD1 is closely related to the adverse clinical outcomes of patients with BC.

## NSD1 displays a high level in paclitaxel-resistant BC cells

**Figure 2.**
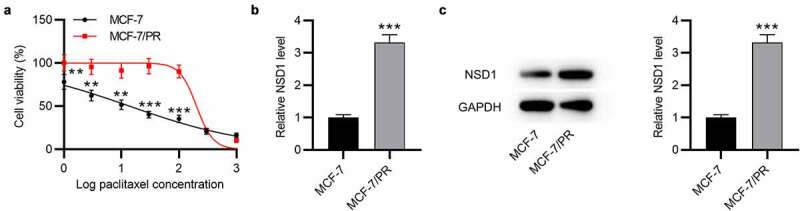
**NSD1 is upregulated in paclitaxel-resistant BC cells**. (a) CCK-8 assay for evaluating cell viability of MCF-7 and MCF-7/PR cells exposed to paclitaxel. (b) RT-qPCR analysis of NSD1 expression in MCF-7 and MCF-7/PR cells. (c) Western blotting of NSD1 protein expression in MCF-7 and MCF-7/PR cells. **p < 0.01, ***p < 0.001

To verify the level of NSD1 in BC, MCF-7 was used to establish paclitaxel-resistant cells (MCF-7/PR). Cell viability was examined in MCF-7 and MCF-7/PR cells. As revealed by the results, MCF-7/PR exhibited higher cell viability than MCF-7 cells after exposure to paclitaxel (Figure 2(a)), indicating that paclitaxel-resistant BC cells were successfully developed. In addition, RT-qPCR and Western blotting demonstrated that NSD1 mRNA and protein expression was upregulated in MCF-7/PR cells in comparison to MCF-7 cells (Figure 2(b-c)).

## NSD1 knockdown suppresses the migration, invasiveness, EMT and paclitaxel resistance of BC cells *in vitr*o

**Figure 3.**
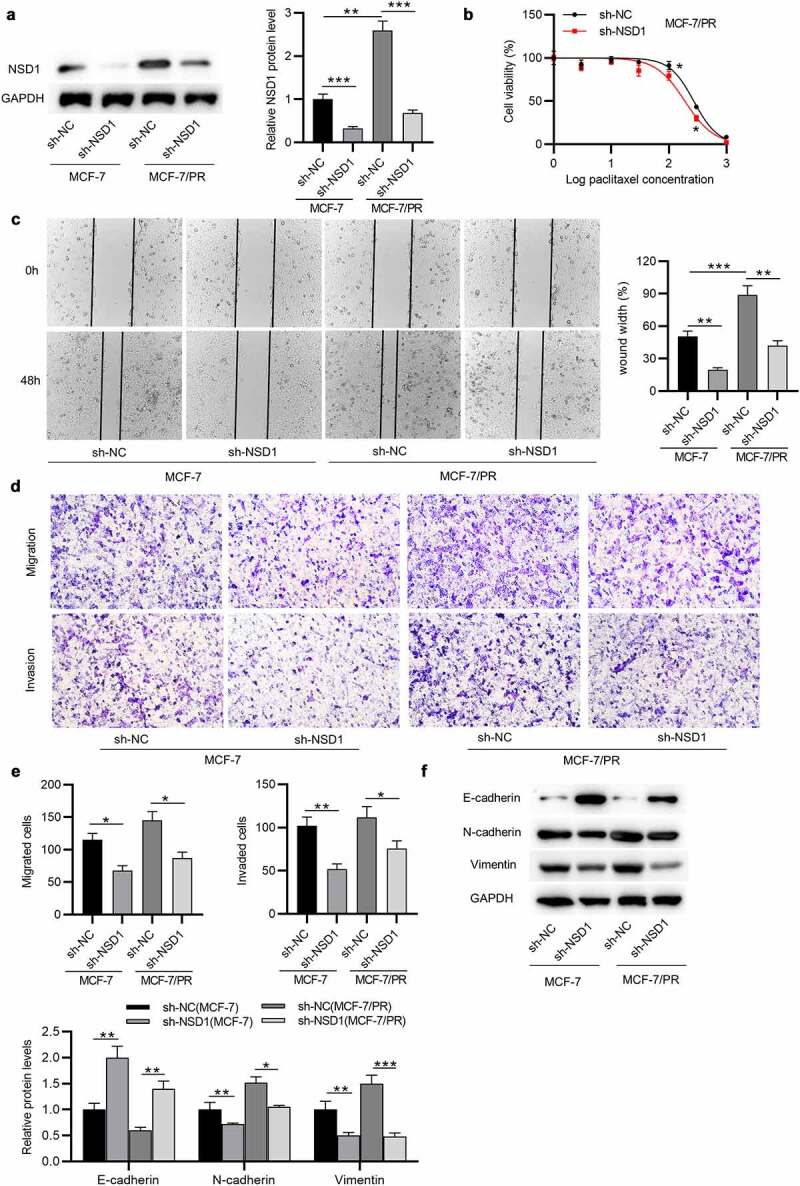
**NSD1 knockdown suppresses the EMT, migration, invasiveness and paclitaxel resistance of BC cells**. (a) Western blotting of the protein expression of NSD1 after knocking down NSD1. (b) CCK-8 assay for analyzing MCF-7/PR cell viability following the interference of NSD1. (c) Wound healing assay of the migratory ability of MCF-7 and MCF-7/PR cells after transfection of sh-NSD1 or sh-NC. (d-e) Transwell assay of the migration and invasion of MCF-7 and MCF-7/PR cells after downregulating NSD1. (f) Western blotting of the protein levels of E-cadherin, N-cadherin and Vimentin in MCF-7 and MCF-7/PR cells with transfection of sh-NSD1 or sh-NC. *p < 0.05, **p < 0.01, ***p < 0.001

To figure out whether NSD1 has an impact on the malignant phenotypes of BC, functional assays were carried out. Western blotting indicated that the NSD1 protein level was decreased after sh-NSD1 transfection in MCF-7 and MCF-7/PR cells (Figure 3(a)). Then, we tested whether NSD1 could impact the sensitivity to paclitaxel in MCF-7/PR cells. As shown by CCK-8 assay, the viability of MCF-7/PR cells in NSD1 silencing group was significantly reduced compared with the control group, (Figure 3(b)), indicating that NSD1 silencing renders MCF-7/PR cells more susceptible to paclitaxel. Moreover, wound healing assay suggested that NSD1 silencing suppressed the migration of MCF-7 and MCF-7/PR cells, and compared with MCF-7 cells, MCF-7/PR cells exhibited greater migratory ability (Figure 3(c)). Similarly, Transwell assay displayed that the number of migrated and invaded cells was significantly reduced after NSD1 downregulation (Figure 3(d-e)). Additionally, as displayed by Western blotting, the protein level of E-cadherin was increased by NSD1 silencing, while that of N-cadherin and Vimentin was markedly reduced (figure 3(f)). Therefore, the above results revealed that NSD1 knockdown promotes paclitaxel sensitivity of BC cells.

## FBXL11 displays a high level in BC and is related to poor prognosis

**Figure 4.**
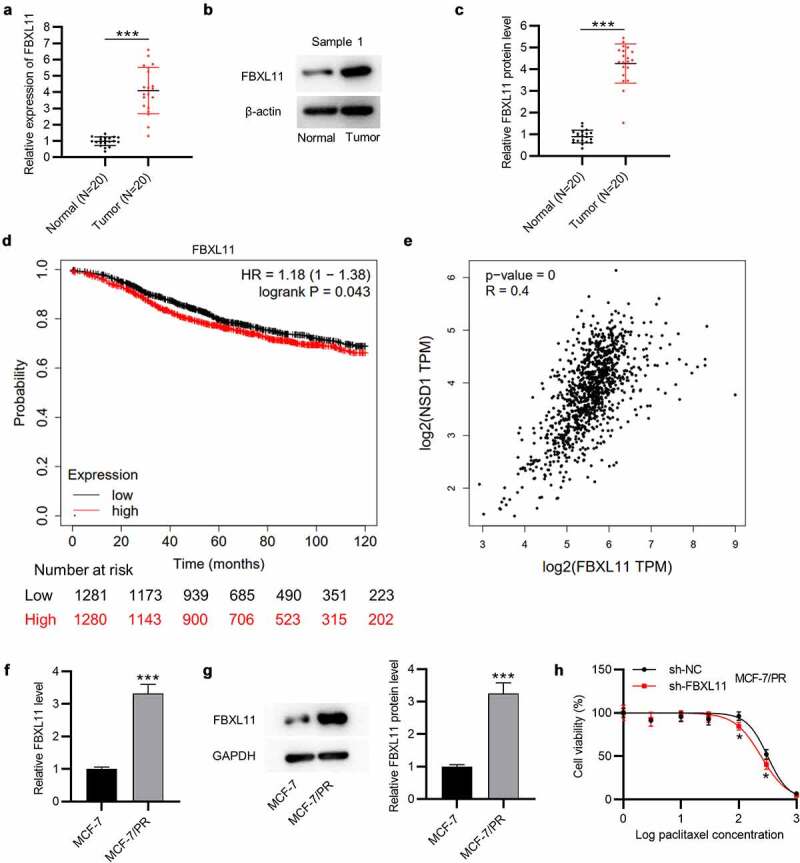
**FBXL11 is upregulated in BC and related to poor prognosis**. (a) RT-qPCR of the expression of FBXL11 in 20 pairs of BC and normal tissues. (b-c) Western blotting of the protein level of FBXL11 is 20 pairs of BC and normal tissues. Protein bands of the other 19 samples are provided in the supplementary file named Figure S1. (d) The relationship between FBXL11 level and BC patients’ prognosis examined by Kaplan-Meier Plotter. (e) The association between the expressions of NSD1 and FBXL11 in BC samples examined by GEPIA database. (f) RT-qPCR analysis of FBXL11 expression in MCF-7 and MCF-7/PR cells. (g) Western blotting of FBXL11 protein expression in BC cells. (h) CCK-8 assay for examining cell viability of MCF-7/PR with FBXL11 silencing. *p < 0.05.***p < 0.001

Furthermore, the expression of FBXL11 at mRNA and protein levels was shown to be upregulated in BC tissues in comparison to corresponding normal tissues (n = 20), as demonstrated by RT-qPCR and Western blotting (Figure 4(a-c)). Data from Kaplan-Meier Plotter show that FBXL11 high level leads to a poor survival probability in patients with BC (Figure 4(d)). Although the difference between FBXL11 high and low levels is not significantly evident, we consider it to be statistically significant as the *p* value is less than 0.05. Moreover, a positive relationship between the levels of NSD1 and FBXL11 in BC samples is observed, as shown by GEPIA database (Figure 4(e)). Compared with MCF-7 cells, the expression of FBXL11 at mRNA and protein levels was elevated in MCF-7/PR cells (figure 4(f-g)). Notably, with exposure to paclitaxel, the viability of MCF-7/PR cells was significantly inhibited following the interference of FBXL11 in comparison to that of the control group (Figure 4(h)). Hence, FBXL11 silencing can promote paclitaxel sensitivity in paclitaxel-resistant cells.

## NSD1 and FBXL11 reversibly regulate NF-kB activity

**Figure 5.**
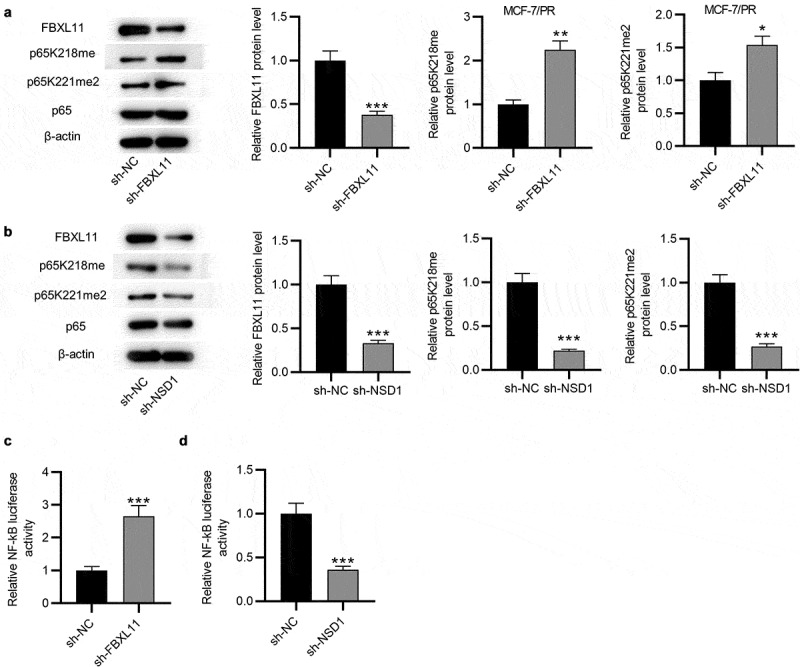
**NSD1 and FBXL11 both regulate NF-kB**. (a) Western blotting was for evaluating the protein levels of FBXL11, p65K218me and p65K221me2 after knocking down FBXL11 in MCF7/PR cells. (b) Western blotting of the protein levels of FBXL11, p65K218me and p65K221me2 after downregulating NSD1. (c-d) The luciferase reporter assay of NF-kB activity after FBXL11 and NSD1 silencing. *p < 0.05, **p < 0.01, ***p < 0.001

Subsequently, we explored the relationship among NSD1, FBXL11 and NF-kB in paclitaxel-resistant BC cells. As shown by Western blotting, after knocking down FBXL11, the protein level of FBXL11 was decreased, while the level of methylation associated proteins (p65K218me and p65K221me2) were markedly increased (Figure 5(a)). This suggested that FBXL11 might exert an inhibitory effect on NF-kB activity. However, NSD1 silencing reduced the protein expression of FBXL11, p65K218me and p65K221me2 (Figure 5(b)), indicating that NSD1 may promote NF-kB activity. Afterward, we suppressed the expression of FBXL11 to detect the luciferase activity of NF-kB, which indicated that FBXL11 knockdown significantly increased NF-kB activity, while NSD1 downregulation had an opposite impact (Figure 5(c-d)). Collectively, these results suggested that the FBXL11/NSD1 pair of demethylase/methylase enzymes reversibly modulates the activity of NF-kB.

## Overexpression of FBXL11 attenuates the inhibitory impact of NSD1 knockdown on the phenotypes of paclitaxel-resistant cells *in vitro*

**Figure 6.**
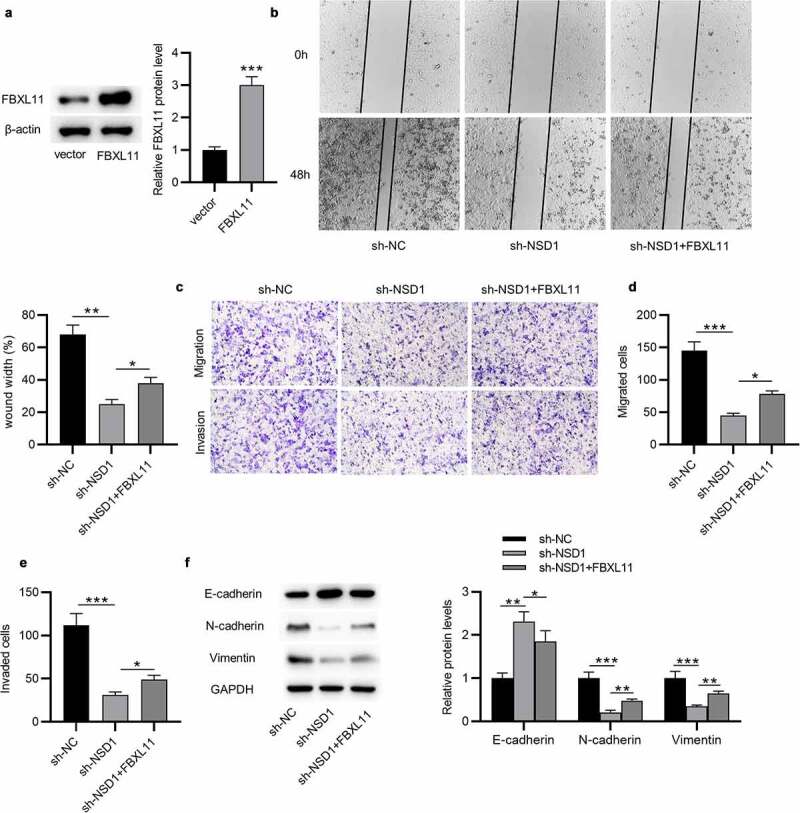
**Overexpression of FBXL11 attenuates the inhibitory impact of NSD1 knockdown on the phenotypes of paclitaxel-resistant cells *in vitro***. (a) Western blotting of the effect of pcDNA3.1/FBXL11 on the protein level of FBXL11. (b) Wound healing assay was utilized for assessing the migratory ability of BC cells transfected with sh-NSD1, sh-NSD1+ FBXL11 or sh-NC. (c-e) Transwell assay of the migratory and invasive capabilities of BC cells with above transfection. (f) Western blotting of the protein levels of E-cadherin, N-cadherin and Vimentin. *p < 0.05, **p < 0.01, ***p < 0.001

Afterward, we explored the effect of FBXL11 in the NDS1-mediated regulation of malignant characters of BC cells. The results of Western blotting demonstrated that the FBXL11 protein level was increased in BC cells after transfection of pcDNA3.1/FBXL11 (Figure 6(a)). Wound healing assay demonstrated that upregulation of FBXL11 abolished the suppressive influence on cell migration caused by NSD1 inhibition (Figure 6(b)). In addition, the migratory and invasive capabilities of BC cells reduced by NSD1 depletion was rescued after upregulating FBXL11, as displayed by Transwell assay (Figure 6(c-e)). Furthermore, the protein level of E-cadherin increased by NSD1 knockdown was partially reversed by FBXL11 overexpression (figure 6(f)). Similarly, the protein levels of N-cadherin and Vimentin reduced by NSD1 inhibition was increased by FBXL11 upregulation, as shown by Western blotting (figure 6(f)). In summary, overexpression of FBXL11 attenuates the inhibitory influence of NSD1 silencing on the malignant behaviors of BC *in vitro*.

## NSD1 knockdown suppresses BC tumor growth and EMT and promotes paclitaxel sensitivity in *vivo*

**Figure 7.**
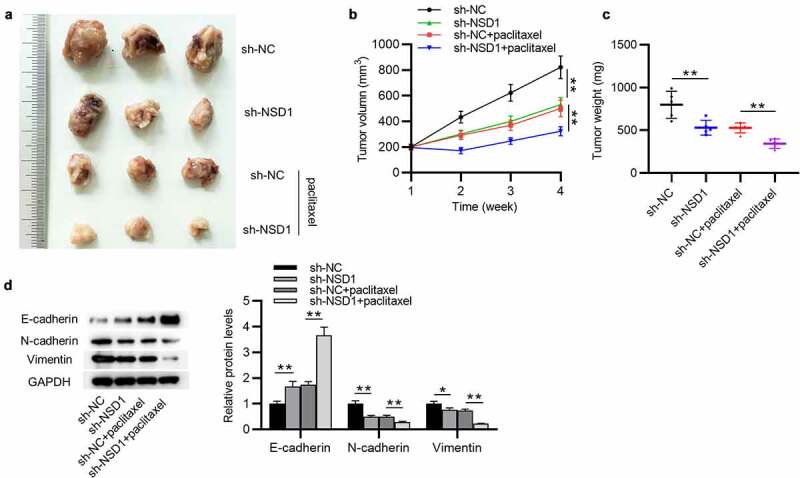
**NSD1 promotes BC growth, EMT and paclitaxel resistance *in vivo***. (a) MCF-7/PR cells transfected with sh-NSD1 or sh-NC were implanted into nude mice (n = 3 each group) by subcutaneous injection. (b) Tumor volumes of every week. (c) The subcutaneous tumor weights at the 28^th^ day after implantation. (d) Western blotting of the protein levels of E-cadherin, N-cadherin and Vimentin in surgically removed tumors. *p < 0.05, **p < 0.01

To further clarify the function of NSD1 in BC, *in vivo* experiment was conducted. MCF-7/PR cells with NSD1 knockdown was injected subcutaneously into nude mice and two groups were treated with paclitaxel (Figure 7(a)). The results of tumor volume curves and tumor weight indicated that NSD1 inhibition significantly suppressed tumor growth in mice (Figure 7(b-c)). Intriguingly, tumors in the sh-NSD1 + paclitaxel group were smaller and grew more slowly than those in the sh-NC + paclitaxel (Figure 7(b-c)), indicating a paclitaxel resistance-inhibitory effect of NSD1 knockdown. Notably, Western blotting analysis indicated that E-cadherin protein levels were elevated in tumors with NSD1 knockdown, while N-cadherin and Vimentin protein expression levels were decreased (Figure 7(d)). All of these results revealed that NSD1 silencing suppresses paclitaxel resistance *in vivo*.

## Discussion

Paclitaxel resistance is a great obstacle in treating BC, and thus multiple studies are focused on finding a solution for paclitaxel resistance. For example, CA12 silencing was reported to facilitate the paclitaxel sensitivity and promote the apoptosis of paclitaxel-resistant BC cells [35]. Here, we investigated the role of a novel gene NSD1 in paclitaxel-resistant BC cells. Additionally, we explored the effects of NSD1 on BC both *in vitro* and *in vivo*. Enhanced NSD1 expression has been observed in a diversity of tumors and data revealed that upregulation of NSD1 is closely associated with the clinical outcomes of patients [10,11,36]. In our study, the mRNA and protein expression levels of NSD1 were upregulated in BC tissues which was verified to have a relation with a poor prognosis. We established a paclitaxel-resistant cell model, and it was found that compared with the sensitive cells (MCF-7), NSD1 displayed a high level in the paclitaxel-resistant cells (MCF-7/PR). Moreover, downregulation of NSD1 repressed the migration, invasiveness and EMT of BC cells *in vitro*. With exposure to paclitaxel, MCF-7/PR cells in sh-NSD1-treated group displayed reduced viability compared with that of sh-NC-treated group, indicating that NSD1 silencing increased the sensitivity to paclitaxel of paclitaxel-resistant BC cells. Additionally, it was confirmed that depletion of NSD1 inhibited BC tumor proliferation, EMT as well as paclitaxel resistance *in vivo*. The results were in accord with previous studies. Hence, NSD1 functions as a tumor promoter in BC.

Emerging evidence has suggested that many messenger RNAs (mRNAs) exert their regulatory functions in cancers by interacting with other mRNAs. For example, CD24 and Hsp70 interact with each other to regulate the progression of lung cancer [37]. To further explore the regulatory mechanism of NSD1 in BC, we detected the mRNA and protein levels of FBXL11 in BC tissues, which was found to be highly expressed. Notably, its elevated expression also reflected a poor prognosis in patients with BC. In addition, FBXL11 also exhibited a high level in MCF-7/PR cells and results showed that FBXL11 silencing promoted paclitaxel sensitivity of MCF-7/PR cells. As shown by GEPIA database, the levels of NSD1 and FBXL11 are positively correlated in BC tissues. It has been reported that upregulation of NSD1 might facilitate oncogenic initiation through reinforced methylation of H3K36 [9]. As reported, NSD1 inhibits H3K27me3 methylation to promote Wnt10b transcription in the development of hepatocellular carcinoma [11]. Moreover, NSD1 has been elucidated to influence methylation of lysine 20 on histone 4 and the non-histone p65 subunit of NF-kB [38]. Here, we investigated whether NSD1 influences NF-kB in a methylation manner in paclitaxel-resistant BC cells. The results revealed that after knocking down NSD1, the protein levels of methylated K218 and K221 of p65 were reduced, indicating that NSD1 functions as a methylase to enhance the activity of NF-kB. Additionally, FBXL11 knockdown in MCF-7/PR cells significantly increased the protein levels of methylated K218 and K221 of p65, indicating that FBXL11 could inhibit NF-kB activity by acting as a demethylase. Results from luciferase reporter assay showed the same effects. These results suggested that the FBXL11/NSD1 pair of demethylase/methylase enzymes reversibly modulates NF-kB activity.

It is recognized that EMT plays a crucial role in drug resistance [39]. For example, the suppression of EMT-inducing transcription factors can enhance the chemotherapy sensitivity of head and neck squamous cell carcinoma cells [40]. Moreover, upregulation of FBXL11 is closely associated with tumorigenesis and lymph node metastasis. For example, FBXL11 facilitates the growth and invasion of cervical tumor cells *in vitro and in vivo* [41]. FBXL11 acts as a tumor activator targeted by miR-29b in gastric cancer [42]. In the current study, overexpression of FBXL11 rescued the inhibitory effects of NSD1 knockdown on the malignant behaviors of BC cells. This effect of FBXL11 is in accord with previous studies. It was shown above that NF-kB, a H3K36 methylase, can activate NF-kB. A previous study has elucidated that expression of the FBXL11 gene is induced on NF-kB activation [17]. Moreover, FBXL11, a histone H3K36 demethylase, was proved to have a negative effect on NF-kB in our study, revealing a negative feedback loop. However, the regulatory effect of NF-kB on FBXL11 was not determined in this study. More studies are demanded to verify whether NF-kB regulates FBXL11 in BC.

## Conclusion

In conclusion, NSD1 facilitates the EMT, migration, and invasiveness in paclitaxel-resistant BC cells by regulating NF-kB and FBXL11. The findings might provide a new prospective for treating paclitaxel-resistant breast cancer.

## Supplementary Material

Supplemental Material

## Highlights

- NSD1 is upregulated in BC and associated with poor prognosis.
- NSD1 knockdown promotes paclitaxel sensitivity of paclitaxel-resistant BC cells.
- NSD1 knockdown suppresses tumor growth *in vivo*.
- NSD1/FBXL11 pair of methylase/demethylase enzymes reversibly regulates NF-kB.
- NSD1 inhibits paclitaxel sensitivity of paclitaxel-resistant BC cells by upregulating FBXL11.

## Abbreviations

BC: breast cancer

NSD1: nuclear receptor binding SET domain protein 1

FBXL11: F-box and leucine-rich repeat protein 11

NF-kB: nuclear factor kappa B

EMT: epithelial-mesenchymal transition

PR: paclitaxel-resistant

DMEM: Dulbecco’s Modified Eagle’s Medium

FBS: fetal bovine serum

RT-qPCR: reverse transcription quantitative polymerase chain reaction

shRNA: short hairpin RNA

PVDF: polyvinylidene fluoride

SD: standard deviation

ANOVA: analysis of variance
