# Supplementary figures and images for: Nuclear receptor binding SET domain protein 1 promotes epithelial-mesenchymal transition in paclitaxel-resistant breast cancer cells via regulating nuclear factor kappa B and F-box and leucine-rich repeat protein 11
Source: Bioengineered. 2021 Dec 14;12(2):11506–19. doi: 10.1080/21655979.2021.2009963 (PMC8810193; doi:10.1080/21655979.2021.2009963)

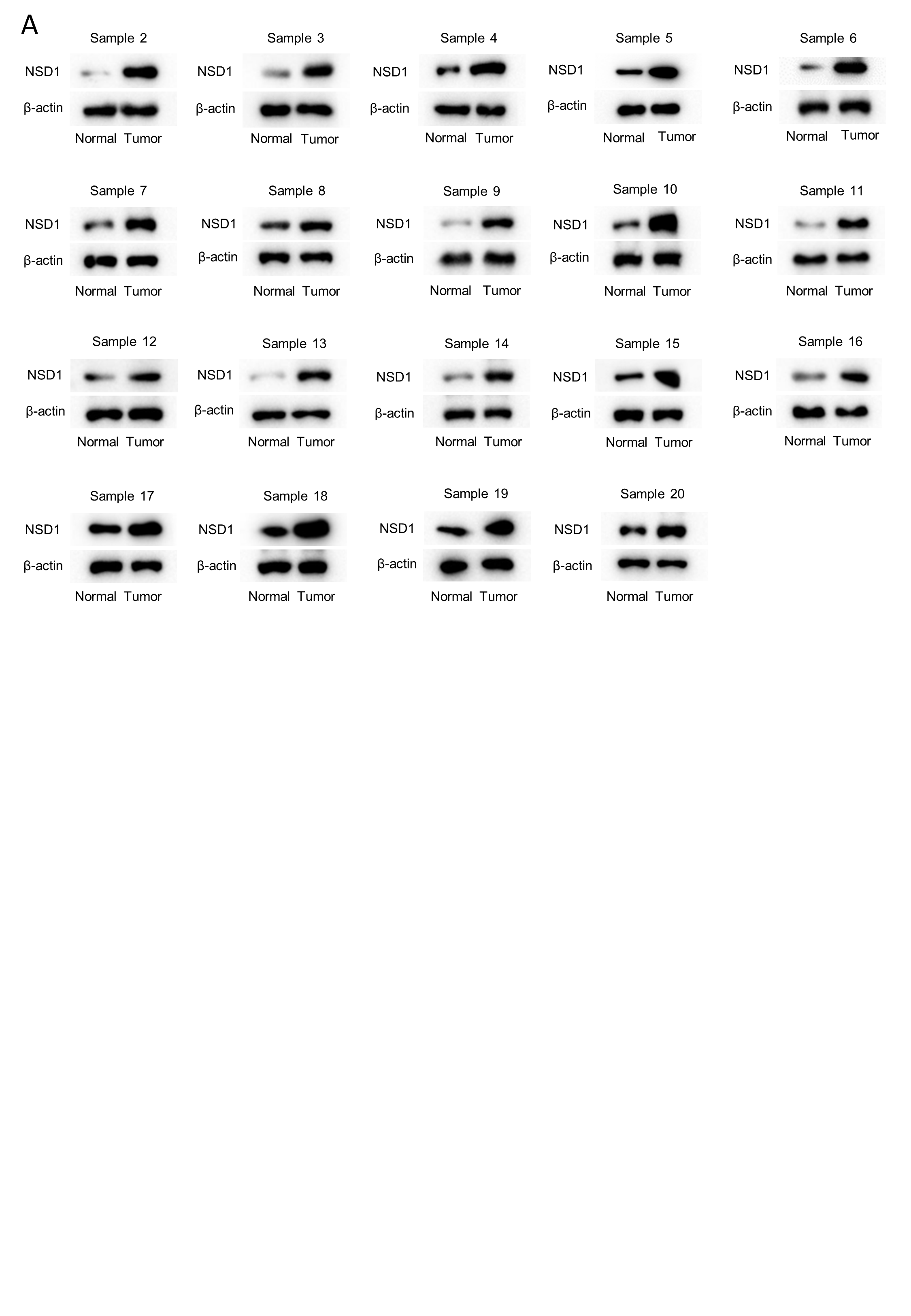

Supplement: Supplemental Material [file KBIE_A_2009963_SM7583.zip › supplementary/Figure S1A.TIF]

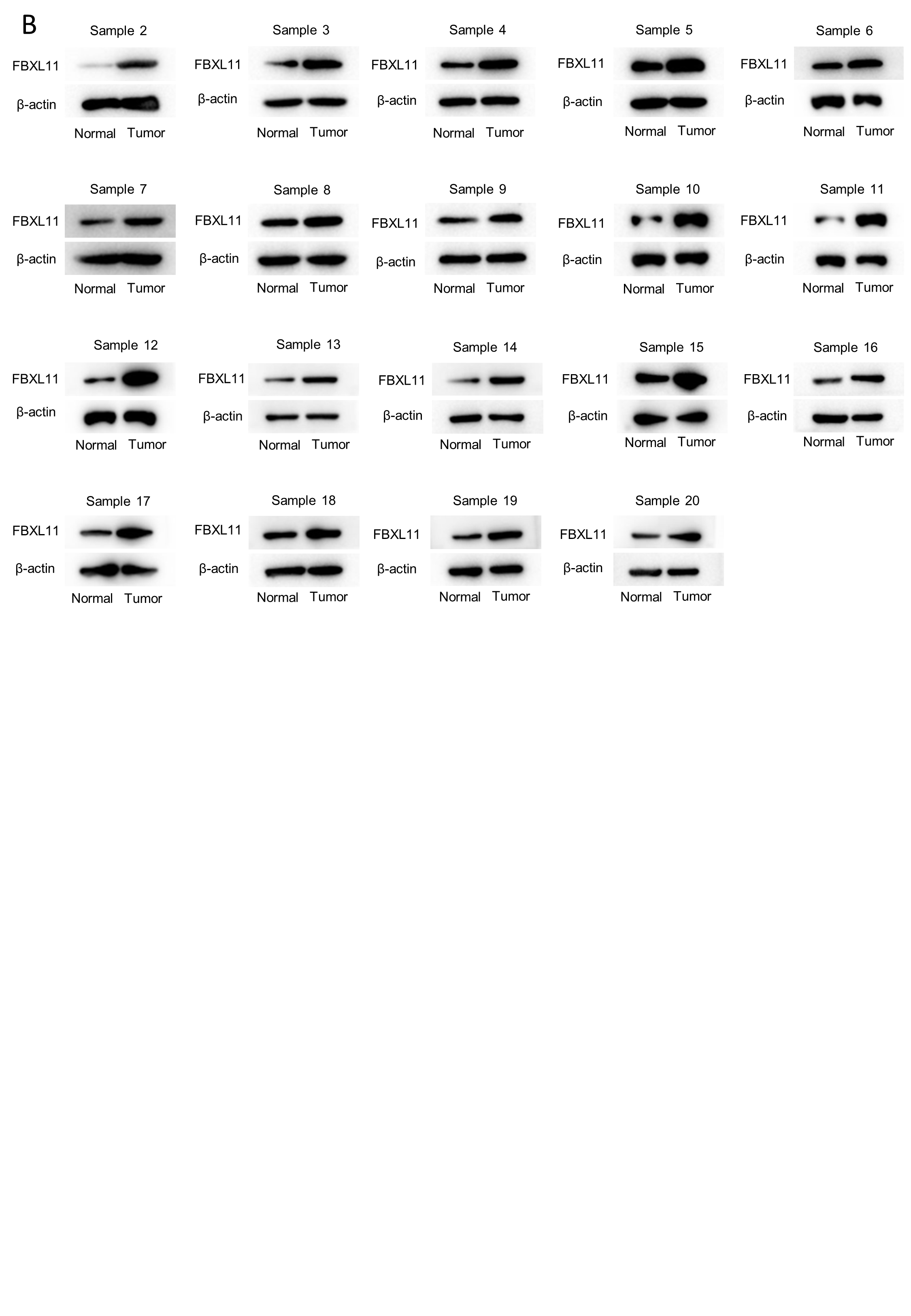

Supplement: Supplemental Material [file KBIE_A_2009963_SM7583.zip › supplementary/Figure S1B.TIF]
